# Two-Dimensional Microstructure-Based Model for Evaluating the Permeability Coefficient of Heterogeneous Construction Materials

**DOI:** 10.3390/ma16175892

**Published:** 2023-08-28

**Authors:** Jiaqi Chen, Shujun Yu, Wei Huang, Hao Wang

**Affiliations:** 1 Department of Civil Engineering, Central South University, Changsha 410075, China; chenjiaqi@csu.edu.cn (J.C.);; 2 Department of Civil and Environmental Engineering, Rutgers, The State University of New Jersey, Piscataway, NJ 08854, USA

**Keywords:** concrete, permeability coefficient, finite element model, ITZ, heterogeneous

## Abstract

The permeability coefficient of construction materials plays a crucial role in engineering quality and durability. In this study, a microstructure model based on real aggregate shape and digital image technology is proposed to predict the permeability coefficient of concrete. A two-dimensional, three-component finite element model of cement concrete was established considering the interfacial transition zone (ITZ) between aggregate and mortar. The permeability coefficient prediction model was developed by the finite element method. The accuracy of the model was verified by experimental data, and the influence of the water−cement ratio on the permeability coefficient of concrete was analyzed. The results show that this method has good prediction accuracy with a relative error of 1.73%. According to the verified model, the influences of aggregate content, aggregate characteristics, aggregate location, ITZ thickness, and other factors on the permeability of concrete were explored. The higher the water−cement ratio, the higher the permeability coefficient. With the increase in aggregate content, the permeability coefficient decreases. Aggregate permeability has a significant influence on the effective permeability coefficient of concrete within a certain range. The greater the roundness of aggregate, the greater the permeability of concrete. On the contrary, the larger aggregate size causes lower permeability. The permeability coefficient of concrete with segregation is lower than that with uniform distribution. At the same time, the permeability increases with the increase of ITZ thickness.

## 1. Introduction

The impermeability of construction materials is a critical requirement for underwater and coastal structures, such as dams, tunnels, and piers. Besides, permeability significantly impacts the durability of concrete structures in cold climates. Lower permeability contributes to enhanced frost resistance and overall durability under such conditions [1,2]. The permeability coefficient is a key material parameter for concrete. The permeability coefficient is affected by several factors, including the inherent properties of concrete, construction techniques, and age [3,4]. The internal pore structure also affects the permeability of concrete materials, especially permeable concrete, which is usually used for facilitating drainage and reducing rainwater runoff [5]. Therefore, effective evaluation of the permeability coefficient of concrete is of great significance to the design, construction, maintenance, and management of concrete structures.

Early studies on the permeability coefficient generally adopted theoretical formulae and laboratory test methods for calculation and analysis. For instance, the typical Katz−Thompson equation [6] established a direct relationship between the permeability coefficient and the porosity and pore characteristics of materials. This equation can be used to calculate the permeability of cement materials with large porosity. However, for ordinary concrete materials with small pore sizes, the accuracy of this equation is sometimes questionable. Meanwhile, the development and application of new materials [7,8,9] may not use these formulae to predict the permeability coefficient accurately. It is widely accepted that the interfacial transition zone (ITZ) between aggregate and mortar is the weak part of concrete [10]. The presence of numerous microcracks within the ITZ contributes to its relatively high porosity. Besides, it contains more calcium hydroxide (CH) [11], a hydration product with low density, which has an important impact on the mechanical properties and permeability of cement concrete. At the same time, some researchers have used the series model [12] and parallel model [13] to predict the permeability coefficient of concrete composite materials. These simplified models are generally efficient and convenient, but it is usually challenging for these models to provide precise predictions. In addition, the general effective medium (GEM) equation [12], finite difference [14], and other numerical calculation methods are often used to derive the concrete permeability coefficient. These methods are common and reliable. The computational cost of these models usually depends on mesh density, especially for heterogeneous models.

Laboratory testing is also a practical method to evaluate the permeability of concrete [15,16,17]. The American Association of State Highway and Transportation Officials (AASHTO) provides various testing methods for measuring the permeability of different concrete materials. Among the various methods available, the hydraulic pressure method is widely employed for determining the permeability coefficient. This method entails monitoring changes in permeation volume and water head over a specific period and subsequently calculating the permeability coefficient. Alternative methods, such as the mercury injection method and water saturation method, are also utilized to evaluate concrete permeability in the existing literature [17]. In addition to the abovementioned methods, researchers have devised test methods to measure the apparent gas permeability of cracked concrete [18] and the permeability of porous media using vacuum technology [19]. These additional methods expand the range of techniques available for assessing concrete permeability.

Although laboratory testing provides direct and accurate data on concrete impermeability, there are some inherent limitations due to variations in testing methods and diversity in testing criteria. For concrete with low permeability, conducting tests often requires the application of high pressure, resulting in long test times. Moreover, it is often difficult to maintain long-term confinement under such high-pressure conditions. Consequently, considering the limitations of experimental testing, numerical simulation methods have been conceived. The advancement of computer technology has facilitated the utilization of CT scanning, digital imaging, nuclear magnetic resonance (NMR) imaging [20,21], and other techniques. These techniques are combined with finite element calculation, allowing for a deeper understanding of concrete microstructure. Scanning electron microscopy (SEM) reveals that the interface region typically has a thickness of 10–50 µm [22,23,24]. Within the above scale, CT scanning is usually costly and time-consuming because high resolution is required when the size of the ITZ is much smaller than that of the whole concrete specimen. To overcome these challenges, some computer algorithm-based virtual random aggregate models [25,26,27] have been developed. Coarse aggregate is an important component of cement concrete, and its influence on permeability is noteworthy. Current studies on coarse aggregate primarily focus on its content in relation to the permeability of concrete [28,29,30]. Most studies on modeling coarse aggregate simplify it as a regular shape, which is somewhat different from the real situation. Therefore, more work is needed to evaluate the permeability coefficients of heterogeneous construction materials, such as concrete.

## 2. Objectives

The main objective of this study is to develop a virtual testing approach for evaluating concrete permeability based on the digital mesostructure of concrete. In this approach, the concrete was simulated as a random medium composed of continuous mortar, discrete aggregates, and an interfacial transition zone (ITZ) between mortar and aggregates. The refined digital aggregates with specific gradations were generated through image analysis and were randomly placed into a digital matrix of mortar. The ITZ between aggregates and mortar was then generated through a digital image processing (DIP) technique. The above digital mesostructure of concrete was imported into the finite element model to compute the permeability coefficient. The accuracy of the virtual testing approach was validated with experimental measurements. Additionally, the effects of ITZ, coarse aggregate content, and mesoscopic parameters of concrete on the permeability coefficient were analyzed with the validated virtual testing approach.

## 3. Model Development

### 3.1. Modelling of Aggregates and Mortar

From the available studies on permeability coefficients, aggregates were generally considered as simpler shapes, such as spheres or ellipsoids, in most finite element simulations of cement concrete [27,28,29]. However, this simplification does not accurately reflect the complexity of actual aggregates, which may lead to deviations in the evaluation of concrete permeability coefficients. For dense concrete, the spatial structure has less influence on its permeability, and the 3-D models require higher computational resources [31]. Therefore, the 2-D models are often preferred for analysis and calculation due to their computational efficiency.

At the microscopic scale, cement concrete is generally regarded as a nonhomogeneous material composed of three distinct components, namely, cement mortar, ITZ, and coarse aggregates [32,33]. In this study, aggregates with particle sizes larger than 4.75 mm were categorized as discrete coarse aggregates. At the same time, aggregates smaller than 4.75 mm were assumed to be part of the continuous cement mortar. This approach could effectively ensure computational efficiency without reducing accuracy. The specific process of establishing the digital model of concrete is as follows.

**Figure 1:**
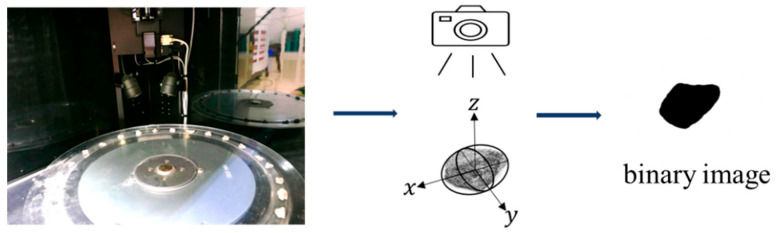
Schematic diagram for generating the 2-D image with AIMS.

To begin, the aggregates with various particle sizes were divided into different groups; 80 representative aggregates were selected randomly to serve as the original aggregate templates. These aggregates were thoroughly washed and dried. After that, the geometry information of these aggregates was collected using the aggregate image system (AIMS), which generated 2-D projections of the aggregates in batches. The resulting projections were processed using image binarization techniques to produce the aggregate template. The experimental setup utilized for this process is shown in Figure 1.

**Figure 2:**
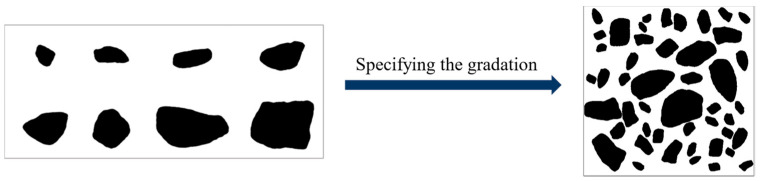
Cement concrete model in PFC 2D.

The aggregate template obtained above was imported into PFC 2D, a discrete element software, and random pellets were generated according to the specified grading using the command flow. Next, the aggregate template was replaced with circles of the same area to achieve a random distribution of coarse aggregate, as shown in Figure 2. The output of the model was saved as a graphic file for further processing.

### 3.2. Modelling of ITZ

It has been found in previous studies that the shape of ITZ is related to many factors, such as aggregate type, particle size, water−cement ratio, etc. [34,35]. Considering that the ITZ only accounts for a small percentage of the total volume of the specimen, in this study, the ITZ was considered to be uniformly wrapped around the exterior of the coarse aggregates.

**Figure 3:**
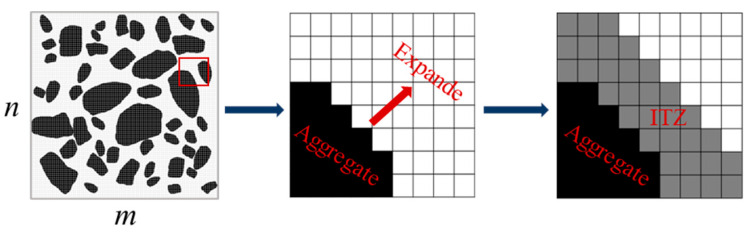
Modelling of ITZ.

To generate ITZ in the digital model, the image files were imported into MATLAB and resized to fit the actual specimen size. The number of discrete pixel points should correspond to the size of the actual model to ensure the uniform thickness of the ITZ. If the size of the actual specimen is *M* × *N*, then the number of pixel points should be *m* × *n*, where *M*:*N* = *m*:*n*. Considering each pixel point as the basic unit of the model, the actual length *x* represented by each pixel point can be obtained through a simple operation, i.e., *x* = *M*/*m* or *x* = *N*/*n*. After discretization, the distance from the aggregate was converted to the ITZ thickness by assigning different colors to the pixels near the aggregate boundary using the dilate function. Figure 3 illustrates the approximate process, where the black area represents the coarse aggregate, the white area represents the cement mortar, and the gray area represents the ITZ. Finally, the processed digital image was converted into a vector image and imported into ABAQUS for modeling calculations.

### 3.3. Calculation of the Permeability Coefficient

**Figure 4:**
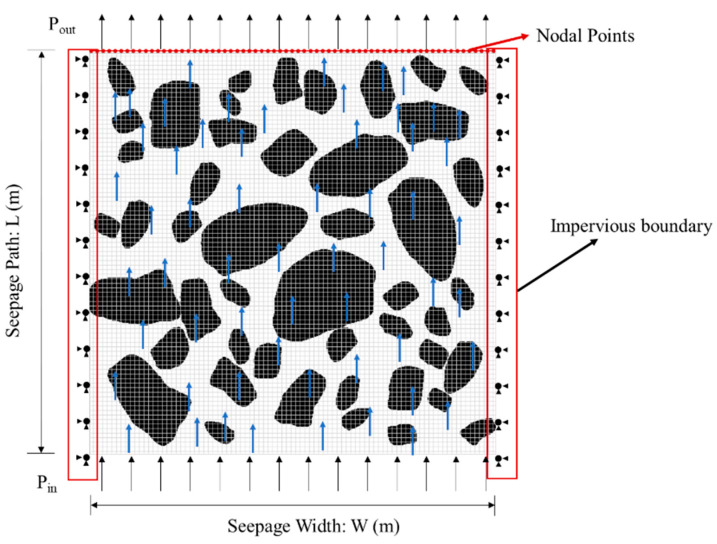
Model calculation diagram.

These FE models focused on the saturated seepage of cement concrete. It assumed the concrete skeleton to be completely rigid, and the water flow was characterized as an incompressible laminar flow. The density of water $\gamma_{w}$ was taken as 9.80 kN/m^3^ in this paper. Therefore, Darcy’s law can be applied to calculate the seepage. The model was discretized into a finite number of cells with meshing, and the flow rate of each node on the water boundary was calculated. The overall flow rate (*Q*) of the concrete specimen can then be determined by summing the flow rate (*Q_i_*) at each node, as illustrated in Figure 4. i.e., $Q=\sum Q_{i}$, then Darcy’s law can be written as Equation (1).

(1) $$K=\frac{\sum Q_{i}L}{A\Delta h}$$

where, *K* is the permeability coefficient of cement concrete, m/s; *Q_i_* represents the flow rate at each node, m^3^/s; ∆*h* is the hydraulic slope, m; *L* is the seepage path, m; *A* is the section area, m^2^. In this paper, *A* = 1 × the width of the specimen *W*.

### 3.4. FE Model Parameters

In the FE model presented in this paper, aggregates with particle sizes smaller than 4.75 mm were considered as part of the mortar, while coarse aggregates ranged in particle size from 4.75 to 25 mm. The aggregate gradation followed the Fuller curve, with cumulative sieve residual data presented in Table 1. Previous research suggested that the thickness of the interface region typically falls within the range of 10–50 µm. Based on previous studies [28,29,36] and to improve simulation accuracy, the thickness of the interface transition region for this paper was set to be 15~20 µm.

According to the characteristics of cement concrete, it was considered that no displacement or deformation occurs in the process of water penetration, and only the seepage field was considered in the numerical simulation. The steady-state analysis was conducted, and all degrees of deformation were constrained in the initial boundary condition, as shown in Figure 4. Impervious boundaries were set on both sides of the model. Furthermore, a hydraulic head gradient is applied at the upper and lower boundaries, corresponding to the FE model’s height in this study.

**Figure 5:**
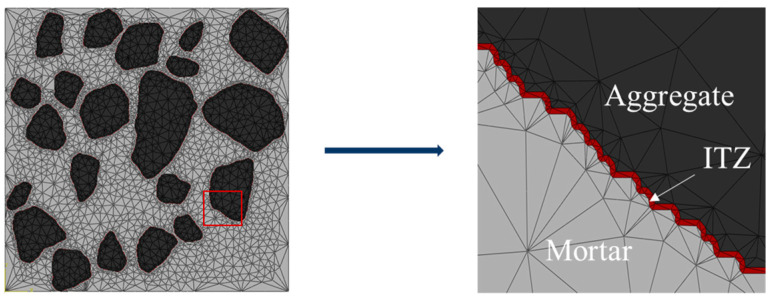
Cement concrete model.

The contact between aggregate, mortar, and ITZ was assumed to be full contact. The geometric discretization employed a quadratic unit, and the model was divided into triangular free grids with a grid size of 5 × 10^−4^ m. Some models were partially encrypted (Figure 5) to ensure normal calculation. In the numerical simulation, the same hydraulic gradient used was applied to the microstructure of cement concrete. The flow rate of the specimen per unit of time was obtained through numerical simulation. This value was substituted into Equation (1) to calculate the permeability coefficient *K*_eff_ of cement concrete.

## 4. Experimental Measurements

### 4.1. Materials

The materials used in this study included PC 42.5 cement, natural sand (apparent density of 2700 kg/m^3^), and limestone aggregate (apparent density of 2720 kg/m^3^). The fine aggregate used was river sand, and the coarse aggregate was limestone with a particle size greater than 4.75 mm. Tap water was added to the mixing process. The round table samples with a base diameter of 175 mm, top diameter of 185 mm, and height of 150 mm were cast. The samples were demolded and subjected to a 28-day curing period under controlled conditions. The curing environment maintained a relative humidity of greater than 95% and a room temperature of 20 °C. Table 1 and Table 2 provide a summary of the concrete mix proportions and coarse aggregate gradation used in the experiment.

### 4.2. Test Method and Results

**Figure 6:**
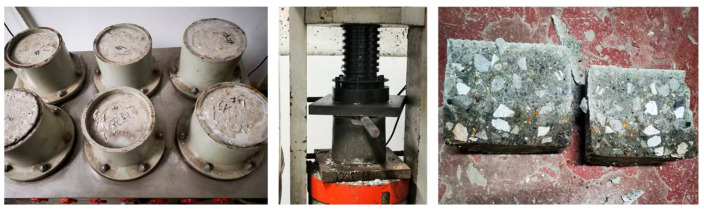
Permeation test equipment and samples.

The seepage height method was used to measure the permeability coefficient of concrete, and the experimental device is shown in Figure 6. The automatic impermeability tester ensured that the water pressure was stable at (1.2 ± 0.05) MPa during the 24-h pressure period. To make all the water penetrate from the inside of the specimens, the specimens must be well sealed. In this experiment, paraffin was employed as a sealing material. Solid paraffin was heated until it melted, and the specimen was immersed in a paraffin basin and carefully rolled back and forth to ensure complete coverage of the entire specimen with paraffin. Subsequently, the paraffin-wrapped specimen was placed within an impermeable instrument mold. To ensure a secure seal, a layer of paraffin wax was poured around the top of the mold, forming a circular seal. After the test, the specimens were split by pressing.

**Figure 7:**
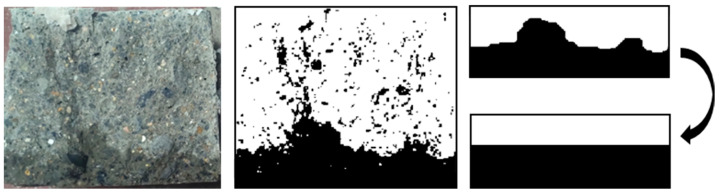
Wetted boundary extraction.

To reduce the measurement error, image processing technology was used to extract the infiltration line and calculate the average height. Firstly, the background of the picture was removed to enhance threshold segmentation accuracy. The corner points of the specimen in the picture were aligned with the corner points of the image, and the size of the image was adjusted to match the actual size, reducing image distortion. Finally, the threshold segmentation method was utilized to extract the infiltrated part. The integral method was applied to calculate the average seepage height *D*, as shown in Figure 7.

(2) $$D=\frac{A}{d}=\frac{\int A_{i}}{d}$$

where, *A* is the total area of the infiltrated part, m^2^, and *d* is the diameter of the specimen, m.

**Figure 8:**
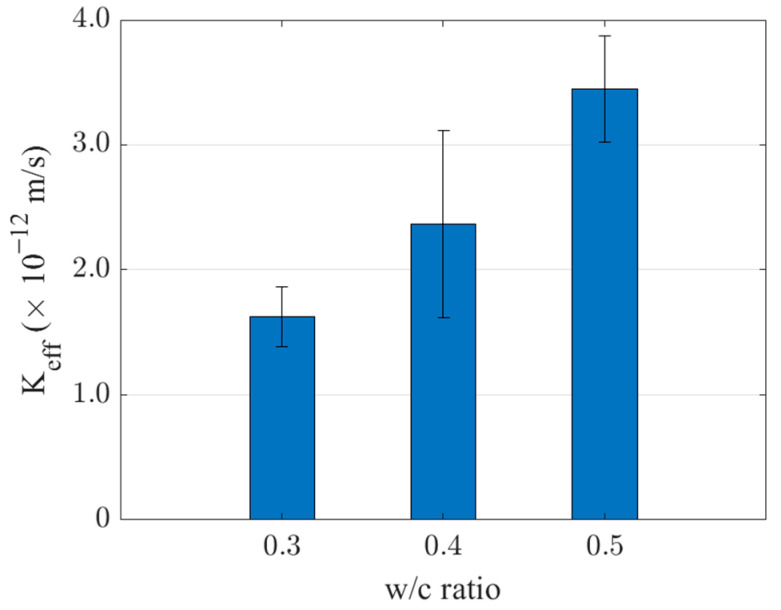
Permeability coefficients of concrete with different w/c ratios.

The permeability coefficient was calculated using Darcy’s Law, Equation (3). The experimental results are presented in Figure 8. It can be observed that the permeability gradually decreases with the increase of w/c ratio. When the water−cement ratio is low, the internal porosity decreases, resulting in denser concrete and a lower permeability coefficient.

(3) $$K_{w}=\frac{\alpha D^{2}}{2TH}$$

where, $K_{w}$ is the concrete permeability coefficient, m/s; *α* is the water absorption rate of concrete, 0.03; *D* is the average seepage height, m; *T* is constant pressure time, 24 h in this experiment; *H* is water pressure, m, 1.2 MPa *=* 1.2 × 10^2^ m.

## 5. Modeling Results and Discussion

### 5.1. Model Validation

In this study, the water−cement ratio was fixed at 0.5. In this case, the permeability coefficient *K*_m_ of cement mortar was set to the average value measured in the experiment at 5.724 × 10^−12^ m/s, and the permeability coefficient of ITZ *K*_i_ was equal to 30 *K*_m_ [28]. According to previous studies, the permeability coefficient of limestone is relatively small, generally ranging from 10^−10^ to 10^−14^ [37,38,39]. In this model, the permeability coefficient of aggregate *K*_a_ was set at 1.0 × 10^−12^ m/s.

Undoubtedly, larger models result in more realistic numerical simulations. However, it is also necessary to consider the effect of the model size on the computation time when dealing with large quantities of models. Due to the significant size difference, it is impractical to model and compute the whole specimen. Consequently, cement concrete models of varied sizes were established to determine the optimal simulation size. The sizes considered include 60 mm × 60 mm, 80 mm × 80 mm, 100 mm × 100 mm and 120 mm × 120 mm. At the same time, based on the above concrete microstructure models, the permeability coefficient of concrete and the calculation time were calculated when the water−cement ratio was 0.5.

**Figure 9:**
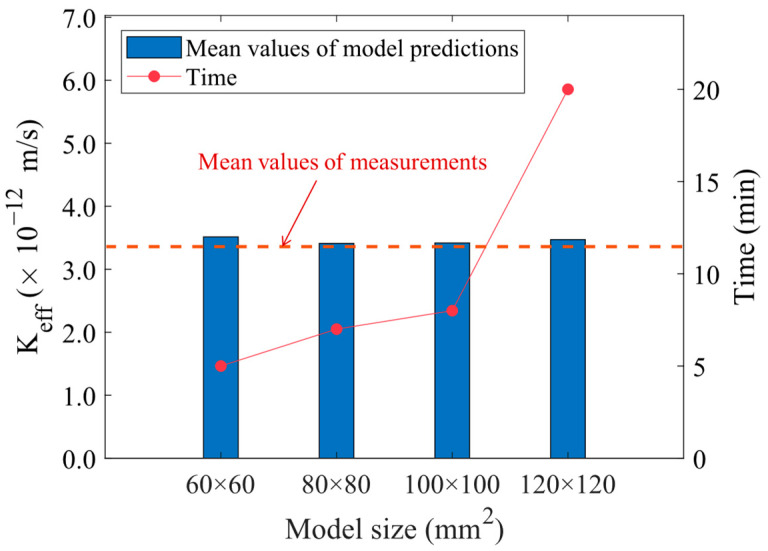
Model validation for different model sizes.

Five values were obtained from different models, and the average permeability coefficient values of the finite element models with different sizes were 3.514 × 10^−12^ m/s, 3.411 × 10^−12^ m/s, 3.417 × 10^−12^ m/s, and 3.471 × 10^−12^ m/s, respectively. The mean value of the measured data was 3.359 × 10^−12^ m/s. The relative errors between the predicted and measured values were 4.61%, 1.55%, 1.73%, and 3.33%, respectively. In general, the accuracy of the permeability calculation model based on microstructure is acceptable. Besides, the computation time was calculated for each model. As shown in Figure 9, the time spent increases exponentially with the increase in model size, while the differences in calculation results are not significant. Therefore, the trade-off between calculation accuracy and computational time was taken into consideration, and the model size was determined to be 100 mm × 100 mm for subsequent analysis.

### 5.2. Effects of Coarse Aggregate Content

**Figure 10:**
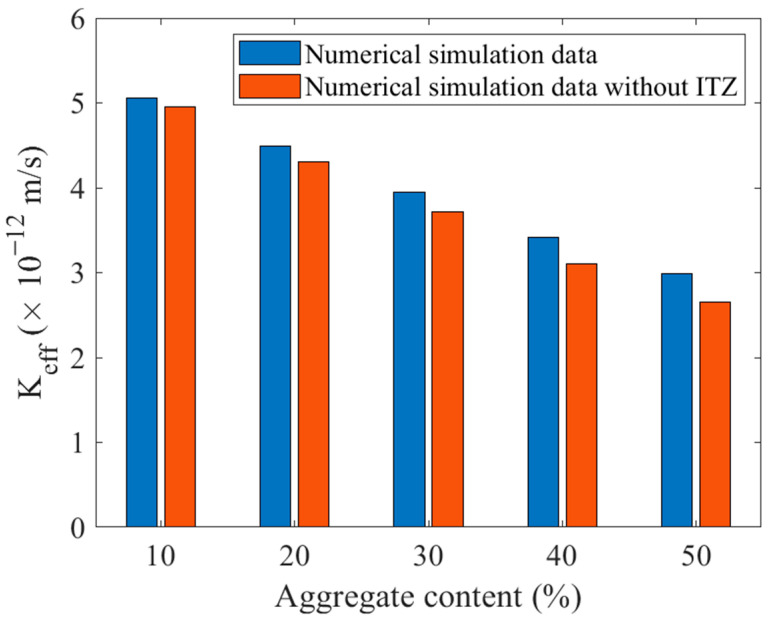
Effects of aggregate content on permeability coefficient.

Based on the finite element microstructure model of cement concrete established with the above method, the permeability coefficients of cement concrete under different aggregate volume contents were calculated. For each content, five different values were obtained from different models. These values were averaged and shown in Figure 10. The permeability coefficient decreases with the increase of aggregate content, on the one hand, because the permeability coefficient of mortar is much larger than that of aggregate, and with the continuous increase of aggregates, the number of tiny pores in concrete becomes less, and the water is difficult to penetrate. On the other hand, the increase of aggregate leads to the water seepage path becoming more tortuous so that the overall permeability coefficient decreases.

At the same time, a total of 25 models without considering ITZ thickness were established for comparison. When ITZ was not considered, the permeability coefficient of concrete was underestimated, especially when the aggregate content was high and the gap was more obvious, which was very unfavorable to the design and normal use of cement concrete with high impervious requirements.

### 5.3. Effects of Aggregate Mesoscopic Parameters

Some studies have concluded that the permeability coefficient of aggregate was very small, which was usually regarded as an impervious material [29]. However, some scholars believe that ignoring the permeability performance of aggregates will cause deviation in the estimation of the overall permeability of concrete [36,40]. At the same time, the permeability coefficient of different aggregates varies greatly [37,38,39]. Therefore, accounting for the potential impact of aggregate permeability, the value cannot be ignored.

**Figure 11:**
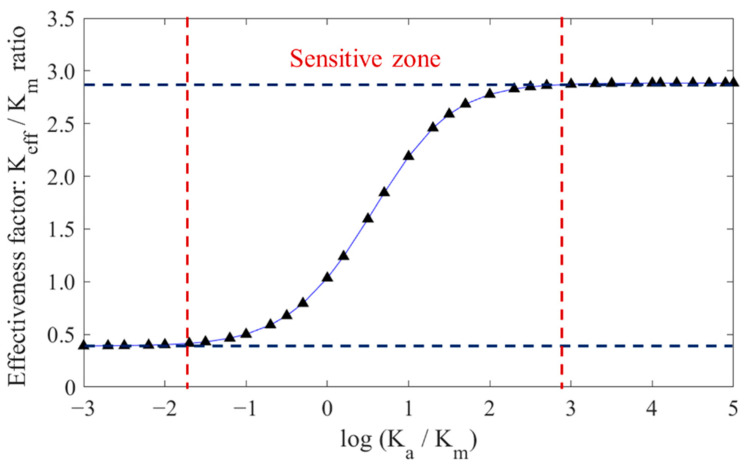
Numerical results of different *K*_eff_/*K*_m_ ratios at different *K*_a_/*K*_m_.

To encompass a wide range of aggregate permeability values in a compact manner, the ratio *K*_a_/*K*_m_ was set from 0.001 to 100,000, and the permeability coefficients of 36 models were calculated. According to previous studies, the w/c ratio of concrete had a great influence on ITZ. Therefore, a fixed water−cement ratio of 0.5 was used when calculating the permeability coefficient of aggregates. The logarithmic coordinate system was drawn according to the above simulation results, as shown in Figure 11. It can be seen that when *K*_a_/*K*_m_ is less than or greater than a certain threshold, *K*_eff_/*K*_m_ basically converges to a constant value. However, within this range, the value of *K*_eff_/*K*_m_ exhibits significant changes with varying *K*_a_/*K*_m_ values, which also reflects that the influence of the aggregate permeability coefficient on the effective permeability coefficient of concrete cannot be ignored within a certain range. The lower and upper limits of the permeability coefficients of aggregates in the sensitive region were determined by interpolating the fitted logarithmic curve, resulting in values of 1.1 × 10^−13^ m/s and 4.5 × 10^−9^ m/s, respectively. The selected parameter for the model in this paper was 1.0 × 10^−12^ m/s, which was located in the sensitive region, indicating the need to consider the permeability of aggregates.

The influence of aggregate shape on the permeability coefficient of cement concrete is an important consideration in practical production, as aggregates can take various shapes. The roundness of aggregates is a parameter that reflects the degree of approximation to a sphere and can affect the permeability of concrete [41]. To evaluate this influence, five groups of digital cement concrete specimens with varying aggregate shapes were established in this study. The roundness of aggregates was quantified using a specific calculation formula, which is presented below.

(4) $$K_{r}=\frac{4\pi A}{L^{2}}$$

where, *K_r_* represents roundness, *A* represents the aggregate area, and *L* represents aggregate contour circumference.

**Figure 12:**
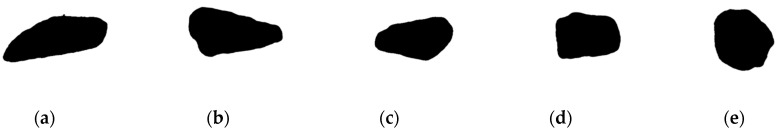
Aggregate models with different roundness (**a**) *K_r_* = 0.5805, (**b**) *K_r_* = 0.6434, (**c**) *K_r_* = 0.7173, (**d**) *K_r_* = 0.7870, (**e**) *K_r_* = 0.8540.

**Figure 13:**
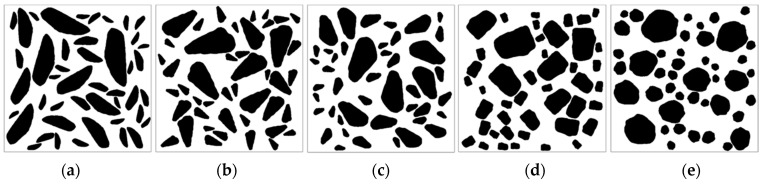
Concrete models with different roundness aggregates (**a**) *K_r_* = 0.5805, (**b**) *K_r_* = 0.6434, (**c**) *K_r_* = 0.7173, (**d**) *K_r_* = 0.7870, (**e**) *K_r_* = 0.8540.

Through Equation (4), the roundness was calculated based on 500 real aggregate templates obtained by AIMS, and five aggregates with different roundness values were selected from them (Figure 12) for subsequent modeling (Figure 13). It is observed that as the value of *K_r_* decreases, the aggregate becomes flatter and longer, while aggregates with larger *K_r_* values tend to be closer to circular in shape.

**Figure 14:**
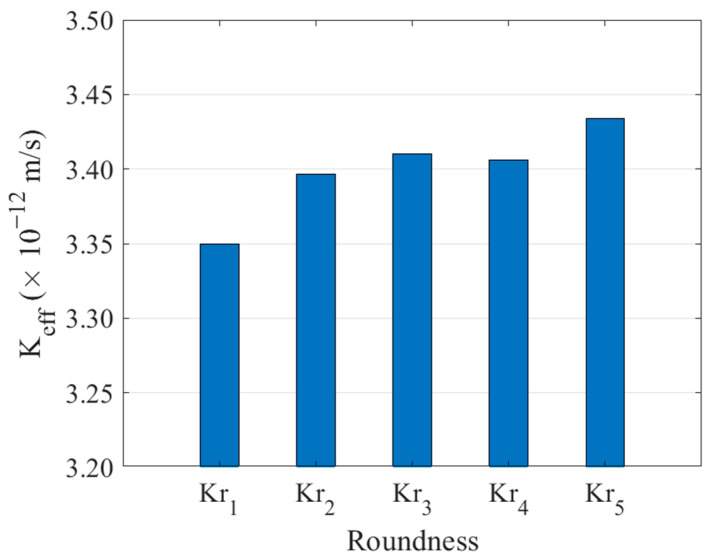
Effects of aggregate roundness on permeability coefficient.

In this study, the numerical simulation method was adopted to calculate the permeability coefficient of the above samples when the aggregate content was about 40%, and the calculation results are shown in Figure 14.

As can be seen from Figure 14, with the increase of aggregate roundness, the permeability coefficient of concrete as a whole shows a slow upward trend. This is because the larger the roundness, the more tortuous the seepage path of water becomes, thus improving the impermeability of concrete.

**Figure 15:**
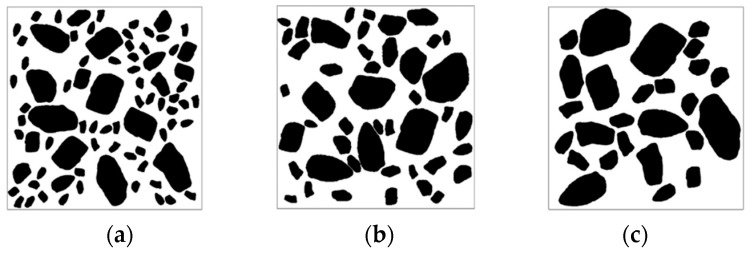
Concrete models with different particle size aggregates (**a**) Smaller particle size, (**b**) Normally distributed particle size, (**c**) Larger particle size.

**Figure 16:**
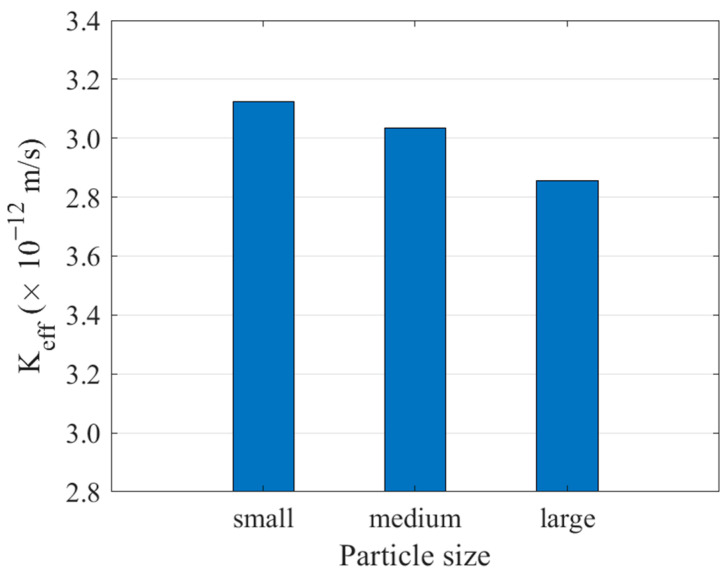
Effects of aggregate particle size on permeability coefficient.

In cement concrete, coarse aggregates serve as a filler to reduce the amount of cement, and their particle sizes vary across a wide grading range. Previous studies [37,39] have demonstrated the significant influence of aggregate particle size on concrete permeability. Consequently, careful consideration should be given to the selection of aggregate particle size during the design and preparation of concrete. In this study, three cement concrete models were established for different aggregate contents, representing the upper and lower limits of the graded particle size and normal size distribution, as depicted in Figure 15. By conducting calculations and averaging the results, the permeability coefficients were obtained, and the findings are presented in Figure 16.

As shown in Figure 16, when the aggregate particle size in concrete is small, the overall permeability of concrete tends to increase. This is because smaller aggregates form more ITZ and provide more permeable channels, resulting in higher permeability of the concrete. At the same time, as the aggregate content in concrete increases, the rate of increase is also more pronounced. Therefore, in the preparation process of concrete, it is crucial to carefully manage the particle size and proportion of aggregates during the concrete preparation process to optimize both the permeability and strength of the concrete.

### 5.4. Effects of Aggregate Segregation

**Figure 17:**
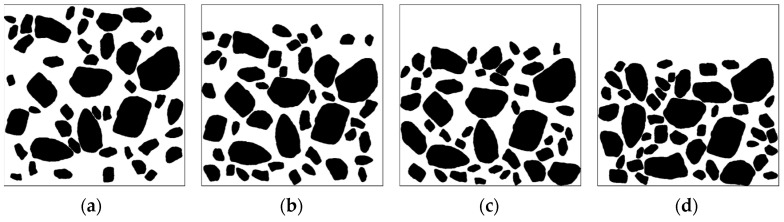
Different degrees of concrete segregation (**a**) 0%, (**b**) 10%, (**c**) 20%, (**d**) 30%.

Studies [42,43,44] have revealed that improper use of the cement water−binder ratio, uneven mixing, and poor curing of specimens can lead to aggregate subsidence and segregation, ultimately affecting the permeability coefficient of concrete specimens. However, in laboratory experiments, it becomes challenging to discern the specific influence of material performance parameters from the effect of aggregate location. Hence, this study did not consider the change in mortar permeability coefficient during segregation. Instead, it focused solely on the effect of aggregate segregation on the concrete permeability coefficient, as shown in Figure 17.

**Figure 18:**
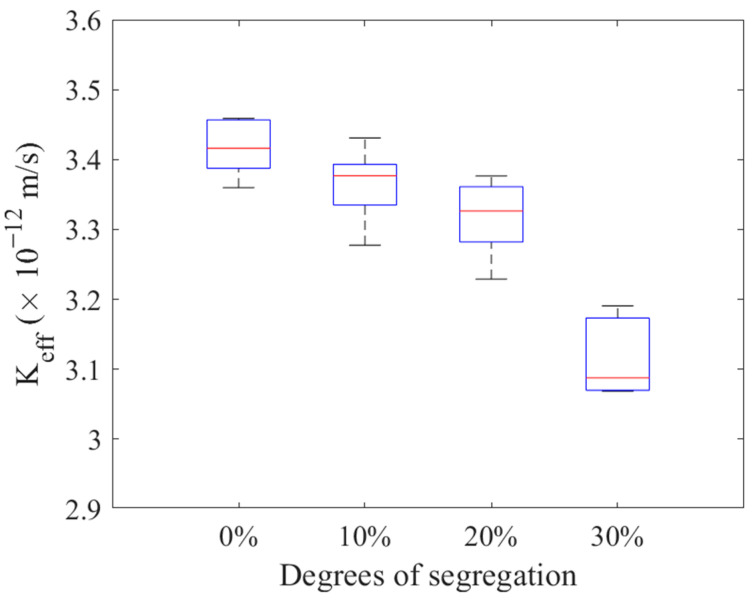
Effects of aggregate segregation on the permeability coefficient.

Four concrete specimens with identical material composition but different segregation conditions were generated, as illustrated in Figure 18. Figure 17a represents the concrete without segregation, where coarse aggregates are uniformly distributed throughout the specimen. On the other hand, Figure 17b–d represents isolated concrete, i.e., 10%, 20%, and 30% of the upper specimens are free of aggregate distribution.

Five models of normal cement concrete and five models of segregated cement concrete were established with numerical simulation, and the permeability coefficients of these samples were calculated at an aggregate content of approximately 40%. The calculation results are presented in Figure 18. It is evident that the permeability coefficient of the concrete with segregation decreased to some extent, and this effect became more pronounced with increasing segregation degree. This can be attributed to the accumulation of aggregates at the bottom of the specimen, which causes the initial water diffusion channels to become more tortuous and affects permeability performance.

### 5.5. Effects of ITZ

**Figure 19:**
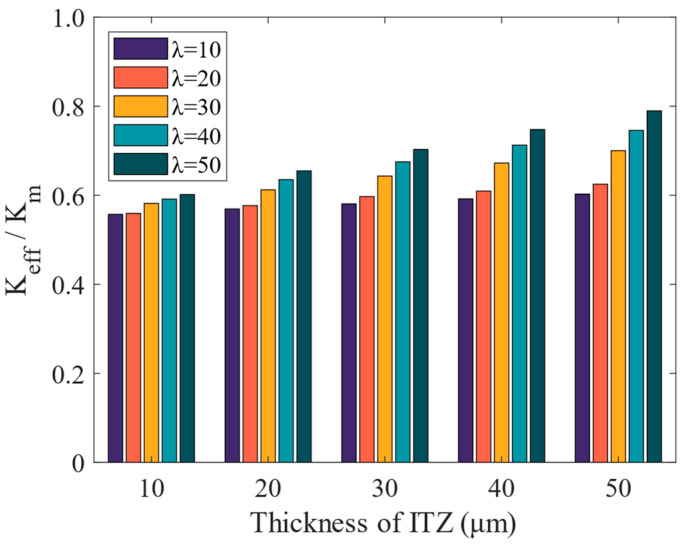
Permeability coefficients of mortar and the effectiveness factor.

The determination of the permeability coefficient of ITZ through laboratory experiments presents challenges due to its particularity. Previous studies have suggested that the w/c ratio of cement concrete and aggregate content are closely related to the permeability coefficient of ITZ [28,29]. To comprehensively investigate the influence of ITZ on the overall permeability of concrete, this study established five concrete models with varying ITZ thicknesses (10, 20, 30, 40, and 50 µm) for sensitivity analysis. The influence of ITZ thickness and permeability on the equivalent permeability coefficient of concrete was studied. It was assumed that the ITZ thickness was uniform and did not vary with aggregate size. The permeability coefficient of ITZ was calculated as *K*_i_ = λ*K*_m_ [29], where λ = 10, 20, 30, 40, 50. The calculation results are presented in Figure 19.

The data presented in Figure 19 indicates that the equivalent permeability coefficient of concrete tends to increase as the thickness of the ITZ increases, with the greatest change observed for ITZ thickness of 50 µm. The increase in ITZ thickness contributes to greater ITZ volume and the possibility of overlapping adjacent coarse aggregate interface areas. This results in the formation of additional ITZ in the pass area, which impacts water penetration. Additionally, the permeable nature of the ITZ itself further influences water penetration. However, the impact of ITZ thickness and permeability coefficient on the entire concrete specimen is relatively minor compared to the influence of aggregate content on the permeability coefficient of concrete. The main reason is the greater volume and distribution of aggregate content in concrete, which is much larger than the content of ITZ. Nevertheless, the thickness of the ITZ is still an important consideration for overall permeability, especially in the design of concrete structures with high durability requirements.

## 6. Conclusions

In this study, computer algorithms and digital image technology are used to establish a 2-D, three-phase microstructure model of cement concrete. The finite element model was used to calculate the permeability coefficient of cement concrete compared to experimental measurement. The following conclusions were drawn from the analysis:

(1) The water−cement ratio of concrete greatly impacts permeability. The experimental results show that the lower w/c ratio leads to more impermeability. The proposed prediction method in this study shows acceptable accuracy, with a relative error of 1.73%.
(2) The permeability coefficient of cement concrete gradually decreases with the increase in coarse aggregate content.
(3) The permeability of aggregate significantly influences the effective permeability coefficient of concrete. The roundness of aggregates has little influence on the permeability coefficient of concrete. However, when the proportion of smaller-sized aggregates is higher, the ITZ increases, leading to a corresponding increase in the permeability coefficient.
(4) The separation of cement concrete leads to the decrease of permeability coefficient. And the decrease is gradual in the 0–20% range, while it becomes more pronounced between 20% and 30%.
(5) With the increase of ITZ thickness, the effective permeability coefficient of concrete increases.

Future study is recommended to develop a 3-D microstructure of concrete and verify the findings of an effective permeability coefficient obtained from 2D models.

## Figures and Tables

**Table 1:** Gradation of concrete.

Sieve size (mm)	25.0	19.0	16.0	9.5	4.75
Passing percentage (%)	100	68.0	48	30	0

**Table 2:** Experimental mix proportions (kg/m^3^).

No.	W/C	Cement	Water	Sand	Gravel
1	0.3	450	150	777	1073
2	0.4	450	180	766	1058
3	0.5	400	200	777	1073
4	0.5	525	263	1477	0

The permeability coefficients of concrete specimens with 40% aggregate content and mortar specimens were measured at water-cement ratios of 0.3, 0.4, and 0.5.

## Data Availability

Data will be made available on request.
